# Case report: Partial visual recovery from incomplete traumatic optic nerve avulsion caused by a badminton shuttle

**DOI:** 10.1016/j.ajoc.2022.101624

**Published:** 2022-06-15

**Authors:** Yuto Kawamata, Yuta Kitamura, Hirotaka Yokouchi, Takayuki Baba

**Affiliations:** a Department of Ophthalmology and Visual Science, Chiba University Graduate School of Medicine, Japan; b Department of Ophthalmology, Kimitsu Chuou Hospital, Japan

**Keywords:** Badminton eye injury, Optic nerve avulsion

## Abstract

**Purpose:**

Blunt ocular trauma rarely results in optic nerve avulsion. Here, we report a case of incomplete optic nerve avulsion caused by the impact of a badminton shuttlecock.

**Observations:**

The patient was a 16-year-old healthy male. A badminton shuttlecock hit his right eye from a short distance. On his first visit to the local eye clinic, his visual acuity in the right eye was hand motion. About 4-mm hyphema in height was observed in the right eye. Three days after the injury, visual acuity improved to 20/50, but the intraocular pressure increased to 40 mmHg; hence, intraocular pressure (IOP)-lowering medication was initiated. Five days after the injury, although hyphema had decreased gradually, he noticed a worsening of vision and was referred to our department. In his right eye, visual acuity was reduced to finger-counting, IOP was 38 mmHg. Slit-lamp examination of the right eye revealed a dilated pupil, hyphema, and angle recession. Fundus examination revealed dilation of the central retinal vein and edematous changes around the optic nerve head. Optical coherence tomography showed a very deep depression of the optic nerve head and partial rupture of the optic nerve axons. B-mode ultrasonography showed hypolucency just posterior to the optic nerve head. Goldmann perimetry revealed a central visual field defect in the right eye. Computed tomography showed no signs of optic canal fracture. These findings suggest that incomplete optic nerve avulsion had occurred. We performed IOP-lowering and anti-inflammatory therapy. After treatment, visual acuity was restored to 20/50, and the deep depression of the optic nerve head recovered to an almost normal range.

**Conclusion and Importance:**

It was assumed that the impact of the badminton shuttlecock caused irreversible changes in the optic nerve head, but the visual function partially improved with IOP-lowering and anti-inflammatory therapy. Because eye injury in badminton can cause severe damage to visual function, every badminton player needs to wear an appropriate eye shield, and rules or guidelines to prevent untoward accidents are needed in badminton.

## 1 Introduction

Badminton is a sport widely enjoyed by men and women of all ages. However, it is not widely recognized that badminton can lead to severe ocular injuries. Approximately 25–40% of ocular trauma requiring hospitalization is sports related.^1^ Badminton is responsible for 14.3% of sports related ocular injuries.^2^ Badminton eye injury can lead to macular degeneration, traumatic cataract, and glaucoma, resulting in poor visual prognosis.^3^ Most badminton-related ocular injuries are due to the impact of the badminton shuttlecock.^3^^,^^4^ Hyphema and angle recession are the most common abnormalities caused by badminton shuttlecock.^5^ Although there have been several reports of direct trauma to the eye caused by the badminton shuttlecock, there have been no reports of damage to the optic nerve. In this report, we describe a case in which a badminton shuttlecock hit the eye, causing visual loss due to incomplete optic nerve avulsion, and partial visual improvement was achieved by intraocular pressure (IOP)-lowering and steroid pulse therapy.

## 2 Case report

**Fig. 1:**
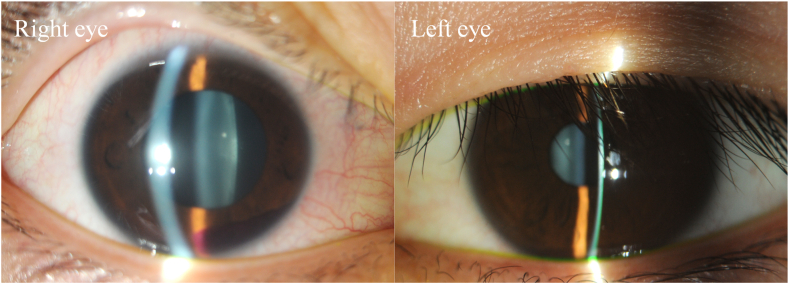
Slit-lamp photography of both eyes. Right eye showed a dilated pupil and mild hyphema. Left eye was normal.

**Fig. 2:**
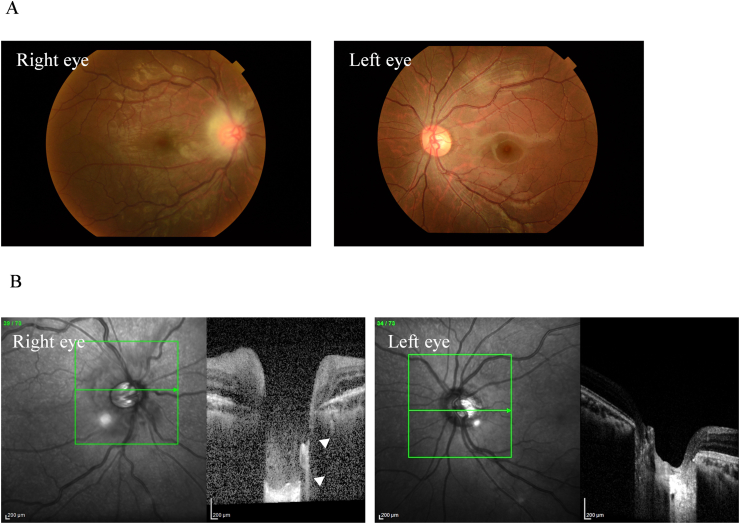
Fundus photograph of the right eye and the left eye showing a slight deformity and edematous change surrounding the optic nerve head. Dilation and tortuosity of the retinal vein are also observed. Fundus of the left eye is normal (A). OCT images of the right eye and the left eye at the first visit showing a deep depression of the optic disc cupping and edema of the optic nerve fiber layer in the right eye. Partial ruptures of the optic nerve axons were suspected (arrowhead) (B).

**Fig. 3:**
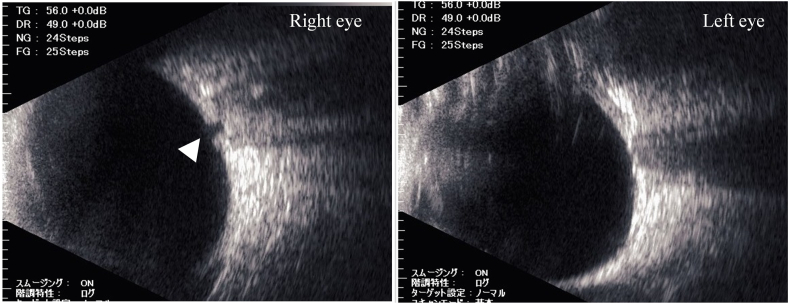
B-mode scan of the right eye and the left eye showing hypolucency (arrowhead) just posterior to the optic nerve head.

**Fig. 4:**
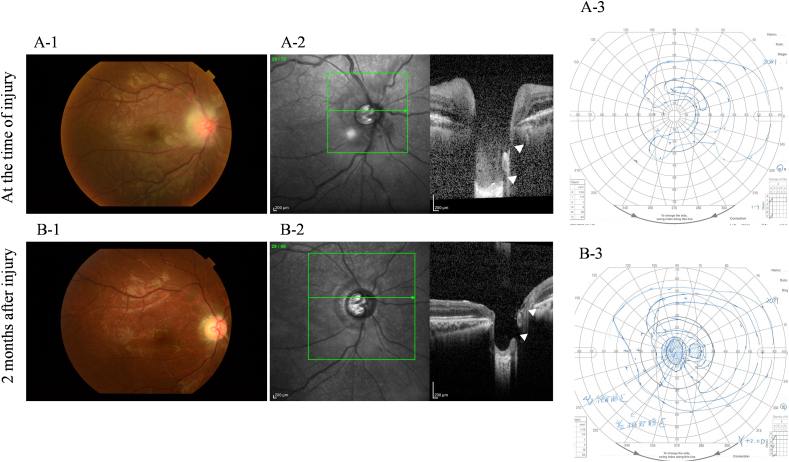
Fundus image of the right eye at the time of injury (A-1) and 2 months after injury (B-1). OCT scan image of the right eye at the time of injury (A-2) and 2 months after injury (B-2). Two months after injury, slight deformity and edematous changes surrounding the optic nerve head disappeared (B-1). Moreover, deep depression of the optic nerve head recovered to almost normal (B-2). Goldmann perimetry revealed a central visual field defect in the right eye at the time of injury (A-3), and partial improvement was observed after treatment (B-3).

**Fig. 5:**
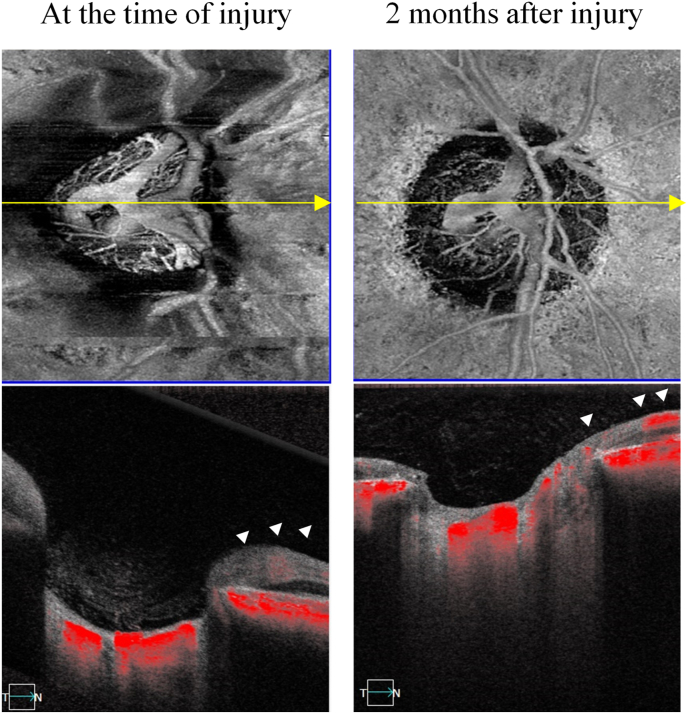
OCT angiography images of the right eye. The images in the left column were taken at the time of injury. The images in the right column were taken 2 months after the injury. Decreased retinal blood flow(arrowhead), dilation of the central retinal vein, and tortuosity of the retinal vein can be observed at the time of injury. Two months after injury, retinal blood flow was restored (arrowhead), and the dilation and tortuosity of the vein were improved.

A 16-year-old male presented to a local eye clinic with a sudden loss of vision in the right eye following blunt trauma with a badminton shuttlecock. He was practicing badminton with senior students over the net. Just after catching the shuttlecock near the net, he was hit by a smash from the other side of the net, which directly hit his right eye at close range (1–2 m). On examination, the visual acuity in the right eye was hand motion. Corneal abrasion and hyphema were observed in the right eye. The amount of hyphema was about 4-mm in height and dense red blood cells were observed in the anterior chamber. The fundus was difficult to observe due to hyphema. Three days after the injury, corneal abrasion disappeared and the amount of hyphema decreased to almost half. Visual acuity improved to 20/50, but IOP increased to 40 mmHg; hence, Carteolol hydrochloride eye drops were started. The patient did not have nausea or vomiting but was aware of mild headache. Five days after the injury, although hyphema decreased to 1-mm in height, the patient experienced a sudden decline in vision in the right eye in the morning, and he was referred to our hospital. He had no medical history of systemic or ocular diseases. On examination at this hospital, his right visual acuity was finger-counting. The IOP in his right eye was 38 mmHg. No apparent injury scars were observed in the right eye. The right relative afferent pupillary defect was unclear because of the dilated pupil, and the average critical flicker frequency (CFF) was 12.8 Hz in the right eye and 41.0 Hz in the left eye. Eye movements were normal. Slit-lamp examination showed a dilated pupil and mild hyphema in the right eye without any apparent injury scars on the conjunctival tissue and eyelid (Fig. 1). Gonial examination showed an angle recession of more than 180°. Fundus examination revealed slight deformities of the optic nerve head and edematous changes in the surrounding area (Fig. 2A). Optical coherence tomography (OCT) showed that the inner retinal layer on the nasal side of the macula was partially thickened and hyperreflective, suggesting circulatory disturbance in the inner retinal layer. Electroretinography (ERG) showed prolonged b-wave latency in the injured eye, suggesting damage to the inner retinal layer. OCT of the optic nerve head showed very deep excavation of the optic nerve head and edema of the optic nerve fiber layer. Furthermore, in some OCT cross-sectional layers, findings suggestive of partial rupture of optic nerve axons were observed (Fig. 2B). FA showed hyperfluorescence of the optic nerve head from the early to the late stages. B-mode ultrasonography revealed hypolucency just posterior to the optic nerve head (Fig. 3). Goldmann perimetry revealed a central visual field defect in the right eye. Computed tomography (CT) and magnetic resonance imaging (MRI) showed no signs of optic canal fractures and deformity or malposition of the optic nerve. These findings suggest that trauma caused by the direct impact of the badminton shuttlecock on the eye may have resulted in incomplete optic nerve avulsion, which in turn caused disturbance of blood and axonal flow around the optic nerve head, resulting in severe impairment of visual function. Although we didn't know if it would be effective, we performed treatment, including IOP-lowering medications to reduce vertical stress on the optic nerve head by reducing static pressure and high-dose systemic steroid therapy for reduction of optic nerve inflammation. We performed these medications in hospitalization care. Specifically, IOP-lowering medications included Tafluprost eye drops, Dorzolamide Hydrochloride/Timolol Maleate combination eye drops, Brimonidine Tartrate eye drops, Ripasudil Hydrochloride Hydrate eye drops, oral acetazolamide 750 mg/day for 7 days, and we also treated with concentrated glycerin intravenous administration at three times. In addition, steroid therapy included 3 doses of high-dose systemic therapy and oral tapering off regimen. Each dose consisted of intravenous methylprednisolone at 1000 mg/day for 3 days, followed by oral prednisolone at 30 mg/day for 4 days. We halved the oral prednisolone every two weeks and cut off on 70 days after we started the treatment. After three doses of steroid pulse therapies followed by oral prednisolone administration, the edematous changes around the optic nerve head disappeared, and the deep depression of the optic nerve head recovered to almost normal range, although rupture of the optic nerve axon remained (Fig. 4). OCT angiography revealed decreased retinal blood flow, dilation of the central retinal vein, and tortuosity of the retinal vein. After treatment, the retinal blood flow was restored, and the dilation and tortuosity of the vein improved (Fig. 5). Visual acuity was restored to 20/50, and partial improvement in visual function was observed.

## 3 Discussion

In this case, a badminton shuttlecock struck the eye at a very high speed at close range, causing optic neuropathy. Based on the findings, incomplete optic nerve avulsion was suspected. There were no bruises or subcutaneous hemorrhages around the eyelid and no subconjunctival hemorrhages. Only corneal damage was observed on the day of injury, suggesting a direct impact on the cornea by a shuttle.

Although badminton is a popular sport among adults and children, the risk of serious eye trauma during badminton play is not enough recognized. A previous report showed that more than half of the cases of badminton eye trauma resulted in chronic vision loss.^3^ Most ocular injuries that occur while playing badminton are caused by a direct hit by the shuttlecock.^3^ Another reported mechanism of eye injury is a direct hit by the racket of a doubles partner.^4^ There are several factors that lead badminton eye injury to serious ocular damage. First, the small size of the shuttlecock base (25–28 mm in diameter) enables entrance into the orbit and has a direct impact on the eyeball. Second, the high velocity of the shuttlecock can reach 400 km/h in an experienced player. Third, the short distance between the players. Especially in doubles, the distance between the players on the same side is short, and there have been reported cases of serious eye injuries caused by a partner's smash in a doubles match.^4^, ^5^, ^6^

Badminton ocular trauma is usually blunt trauma. In blunt trauma, the cornea and sclera are suddenly compressed at the moment of impact causing a compensatory expansion of the equatorial region of the globe. As a result, patients present with hyphema, traumatic cataract, subluxation of the lens, sphincter tear, cyclodialysis, iridodialysis, vitreous hemorrhage, Berlin's edema, retinal detachment, choroidal rupture, and secondary glaucoma.^3^^,^^4^^,^^6^^,^^7^ In this way, non-penetrating ocular injuries can result in severe ocular damage and significant loss of vision.^8^ Traumatic hyphemia is one of the most common ocular's presentations of eye injury in badminton. It occurs due to rupture of the vessels of the iris and ciliary body by blunt force to the orbit. Hyphemia can be followed by increased IOP due to the occlusion of the trabecular meshwork by blood clots and inflammatory cells. In our case, hyphemia and secondary IOP elevation were observed. We speculate that the partial improvement in visual acuity on the third day of injury was due to the improvement of the hyphema and corneal abrasion that occurred immediately after the injury. Subsequently, secondary IOP elevation loaded high vertical stress on the optic nerve head area which had become more vulnerable due to traumatic optic nerve avulsion. This vertical stress placed a load on the cribriform plate to move it further posteriorly, exacerbating the retinal circulatory failure and secondary glaucomatous axonal damage, which is assumed to have reduced vision on the fifth day of the injury.

Optic nerve avulsion is a rare complication of ocular trauma.^8^ There have been no reports of optic nerve avulsion due to ocular trauma in badminton players. It is most often caused by direct dissection or rupture associated with open globe injury, but there are some cases of indirect onset associated with blunt trauma.^9^^,^^10^ Traumatic optic nerve avulsion is thought to involve the optic nerve being pushed out of the scleral canal by the sudden extreme rotation of the eye, moving it forward, and by a sudden increase in IOP.^8^ It is thought to cause tearing of the unmyelinated nerve fibers of the optic nerve near the junction of the eye and optic nerve, especially in the cribriform plate region, which is structurally fragile plate region. Cirovic et al. conducted a computer modeling study to reveals the mechanism of blunt eye injury and its effect on the eye. They were assumed to impact the eyes at a speed of 2–5 m/s. The simulation results showed that the maximum IOP reached 300 mmHg instantaneously, and the stress of the impact was concentrated in the area where the optic nerve inserts into the sclera and lamina cribrosa due to eye rotation and high IOP.^11^

The diagnosis of optic nerve avulsion is based on the observation of a deep excavation of the optic disc or disappearance of the optic nerve on fundus examination. OCT is also a very useful test for observing deep depressions in the optic nerve head. However, it is often difficult to diagnose because of vitreous or anterior chamber hemorrhage. Sawhney et al. reported that B-scan ultrasonography was effective in diagnosing optic nerve avulsion in cases where the disc area could not be observed.^8^ They reported that hypolucency just posterior to the optic nerve head was observed. In our case, although no signs of optic nerve avulsion were suggested on CT or MRI scans, OCT showed not only a very deep depression of the optic nerve head, but also partial rupture of the optic nerve axon. Moreover, hypolucency immediately posterior to the optic nerve head was observed on B-scan ultrasonography. Based on this evidence of impaired vision and lowered CFF values, we suspected that incomplete optic nerve head avulsion had occurred. Although mechanical damage to the optic nerve and supporting tissues at the time of trauma was considered unlikely to improve, we performed symptomatic treatment, including IOP-lowering drops and oral acetazolamide administration to reduce vertical stress on the optic nerve head and three steroid pulse therapies for anti-inflammatory effects on the optic nerve. After treatment, the visual acuity improved to 20/50, and deep depression of the optic nerve head recovered to an almost normal range. This may reflect the reversal of lamina cribrosa displacement due to the lowering of the IOP. The mechanism by which the optic nerve head depression is restored is not clearly understood. Lee et al. reported that the reversal of lamina cribrosa displacement occurred after trabeculectomy in glaucoma patients.^12^ They also reported that younger age and greater IOP reduction were significantly associated with anterior lamina cribrosa movement after surgery. In our case, the patient was young and the IOP reduction rate was high (60% at 2 months after treatment). The high rate of IOP reduction in young patients may be influenced by the high elastic recovery of connective tissue around the lamina cribrosa. Blunt eye injury can cause late-onset macular edema, macular degeneration, optic atrophy, and angle-recession glaucoma.^6^ Late onset angle recession glaucoma can occur anytime even years after the injury.^13^Ng et al. reported that angle recession glaucoma occurred more than 5 years after the injury.^14^ Several studies revealed cases with more than 180° of angle recession are more likely to develop late onset glaucoma.^14^^,^^15^ In our case, the degree of angle recession was more than 180°. Therefore, periodical follow-up by an ophthalmologist is needed even several years after the injury. Tesluk et al. reported that 55% of angle recession glaucoma patients developed open-angle glaucoma in the non-traumatized contralateral eye. According to their research, follow up for the fellow eye is also necessary. We educated the patient about the risk of late onset angle recession glaucoma and informed not to quit receiving regular outpatient medical treatment.^16^ Finally, we mentioned the inadequacy of dissemination activities regarding the prevention of ocular trauma in badminton. Badminton requires quick movements and high-quality kinetic vision, and the need to ensure visibility during play has prevented the widespread use of goggles. It is desirable to raise awareness of the risk of serious eye trauma in badminton and to advocate protective use of goggles that do not affect play. In the Canadian province of Ontario, all junior double players are mandated to wear protective eyewear in competitive games that meet the international American Society for Testing and Materials (ASTM)F803 standard because of recent cases of eye trauma that have occurred while playing badminton. British Columbia also mandated F803 eyewear for players younger than 19 years old.^13^

## 4 Conclusion

We encountered a case of incomplete optic nerve avulsion due to badminton shuttlecock eye injury.

Although badminton eye injuries can cause severe damage to visual function, there is insufficient awareness of the need for eye protection. Rules or guidelines are needed in badminton so that players can wear appropriate eye shields.

## Patient consent

Consent to publish the case report was not obtained. This report does not contain any personal information that could lead to the identification of the patient.
